# S100A8/A9, a potent serum and molecular imaging biomarker for synovial inflammation and joint destruction in seronegative experimental arthritis

**DOI:** 10.1186/s13075-016-1121-z

**Published:** 2016-10-24

**Authors:** Edwin J. W. Geven, Martijn H. J. van den Bosch, Irene Di Ceglie, Giuliana Ascone, Shahla Abdollahi-Roodsaz, Annet W. Sloetjes, Sven Hermann, Michael Schäfers, Fons A. J. van de Loo, Peter M. van der Kraan, Marije I. Koenders, Dirk Foell, Johannes Roth, Thomas Vogl, Peter L. E. M. van Lent

**Affiliations:** 1 Experimental Rheumatology, Radboud University Medical Center, PO Box 9101, 6525 GA Nijmegen, The Netherlands; 2 European Institute for Molecular Imaging, University of Münster, Münster, Germany; 3 Department of Pediatric Rheumatology and Immunology, University of Münster, Münster, Germany; 4 Institute of Immunology, University of Münster, Münster, Germany

**Keywords:** S100A8/A9, IL-1Ra, Seronegative arthritis, Biomarker, Imaging, Cartilage damage, MMP, Inflammation

## Abstract

**Background:**

Seronegative joint diseases are characterized by a lack of well-defined biomarkers since autoantibodies are not elevated. Calprotectin (S100A8/A9) is a damage-associated molecular pattern (DAMP) which is released by activated phagocytes, and high levels are found in seronegative arthritides. In this study, we investigated the biomarker potential of systemic and local levels of these S100 proteins to assess joint inflammation and joint destruction in an experimental model for seronegative arthritis.

**Methods:**

Serum levels of S100A8/A9 and various cytokines were monitored during disease development in interleukin-1 receptor antagonist (IL-1Ra)^–/–^ mice using ELISA and multiplex bead-based immunoassay, and were correlated to macroscopic and microscopic parameters for joint inflammation, bone erosion, and cartilage damage. Local expression of S100A8 and S100A9 and matrix metalloproteinase (MMP)-mediated cartilage damage in the ankle joints were investigated by immunohistochemistry. In addition, local S100A8 and activated MMPs were monitored in vivo by optical imaging using anti-S100A8-Cy7 and AF489-Cy5.5, a specific tracer for activated MMPs.

**Results:**

Serum levels of S100A8/A9 were significantly increased in IL-1Ra^–/–^ mice and correlated with macroscopic joint swelling and histological inflammation, while serum levels of pro-inflammatory cytokines did not correlate with joint swelling. In addition, early serum S100A8/A9 levels were prognostic for disease outcome at a later stage. The increased serum S100A8/A9 levels were reflected by an increased expression of S100A8 and S100A9 within the ankle joint, as visualized by molecular imaging. Next to inflammatory processes, serum S100A8/A9 also correlated with histological parameters for bone erosion and cartilage damage. In addition, arthritic IL-1Ra^–/–^ mice with increased synovial S100A8 and S100A9 expression showed increased cartilage damage that coincided with MMP-mediated neoepitope expression and in vivo imaging of activated MMPs.

**Conclusions:**

Expression of S100A8 and S100A9 in IL-1Ra^–/–^ mice strongly correlates with synovial inflammation, bone erosion, and cartilage damage, underlining the potential of S100A8/A9 as a systemic and local biomarker in seronegative arthritis not only for assessing inflammation but also for assessing severity of inflammatory joint destruction.

**Electronic supplementary material:**

The online version of this article (doi:10.1186/s13075-016-1121-z) contains supplementary material, which is available to authorized users.

## Background

Seronegative arthritides are a large heterogeneous group of joint diseases, which include spondyloarthritic diseases (SpA; e.g. ankylosing spondylitis (AS) and psoriatic arthritis (PsA)) and juvenile idiopathic arthritis (JIA), amongst others. A common feature in these patients is the lack of increased levels of serum autoantibodies, such as rheumatoid factor (RF) and anti-citrullinated protein antibodies (ACPA) [1–4], the presence of which is a typical autoimmune feature in rheumatoid arthritis (RA) patients. While in RA patients the serum RF and ACPA levels, together with erythrocyte sedimentation rate (ESR) and C-reactive protein (CRP) levels, are established serum biomarkers to assess disease activity, only ESR and CRP are used as a serum biomarker in seronegative arthritic patients for appropriate patient management and treat-to-target strategy.

Currently, a considerable number of studies are exploring the potential of new diagnostic and predictive markers for several forms of seronegative arthritis, including calprotectin (S100A8/A9) [5–7]. Whereas in RA serum S100A8/A9 has been widely accepted as a powerful serum biomarker to assess disease activity and to predict therapy response [8], its role as a serum biomarker in seronegative arthritis is still under investigation [9–12]. S100A8 (MRP8) and S100A9 (MRP14) are calcium-binding proteins which belong to the group of damage-associated molecular patterns (DAMPs) or alarmins and are selectively expressed in phagocytes, i.e., granulocytes, monocytes, and activated macrophages. Both proteins are co-expressed and form a stable heterodimer S100A8/A9, which is the predominant occurring form and able to activate macrophages via binding and activation of Toll-like receptor (TLR)4-dependent signaling cascades [13]. Although human and murine S100A8 and S100A9 show limited sequence similarity, the tertiary structure is very similar and both human and murine S100A8, S100A9, and S100A8/A9 have been shown to bind to TLR4 [14–18].

S100A8 and S100A9 have been shown to induce chemotaxis and transendothelial migration of phagocytes to the inflamed tissue and to stimulate the release of pro-inflammatory cytokines and chemokines from activated macrophages [13]. Next to these inflammatory processes, these DAMPs have also been implicated in joint damage in arthritic diseases. S100A8 and S100A9 were found to directly stimulate matrix metalloproteinase (MMP) expression in murine and human chondrocytes, thereby facilitating the breakdown of cartilage in RA and osteoarthritis [19–21]. Indeed, S100A9 has been shown to regulate inflammation and cartilage destruction, and to be a promising imaging biomarker tool to assess disease severity in experimental arthritis models [22, 23].

These findings indicate that S100A8 and S100A9 may be relevant biomarkers for inflammatory processes as well as processes involved in joint destruction. Indeed, serum S100A8/A9 levels are associated with several radiographic joint damage scores in RA (i.e., modified Sharp score and RA Articular Damage score) [8, 24], but the biomarker potential of serum S100A8/A9 for joint destruction is less clear in seronegative arthritis [25, 26].

In this study, we explore the biomarker potential of S100A8/A9 in interleukin-1 receptor antagonist deficient (IL-1Ra^–/–^) mice, a non-immune complex-mediated arthritis model. In these mice, the deficiency of IL-1Ra leads to increased IL-1 signaling and subsequent spontaneous development of inflammation in the hind paws which will eventually lead to cartilage and bone destruction [27]. Increased IL-1 signaling has also been found in several types of seronegative arthritides [28, 29], but is most pronounced in systemic onset juvenile idiopathic arthritis (sJIA) [30]. Although low levels of autoantibodies against immunoglobulin (Ig)G, type II collagenase and double-stranded DNA are formed in IL-1Ra^–/–^ mice, these autoantibody levels do not correlate with disease severity, and even mice with low autoantibody levels developed arthritis [27]. Clearly, the IL-1Ra^–/–^ mouse may not be a model for AS, PsA, or JIA since these conditions are very heterogeneous and many of the clinical manifestations are not shared by IL-1Ra^–/–^ mice. However, IL-1Ra^–/–^ mice may be a relevant model to explore new biomarkers for arthritides in which serum autoantibodies are not increased.

In this study, we explored the potential of S100A8/A9 as a systemic and local biomarker (monitored by ELISA and in vivo imaging) for joint inflammation, bone erosion, and MMP-mediated cartilage damage in IL-1Ra^–/–^ mice.

## Methods

### Animals

Male and female IL-1Ra^–/–^ mice from the BALB/c background were kindly provided by Dr. M. Nicklin (Sheffield, UK) and were generated as described previously [31]. Male and female BALB/c control mice (Janvier, France) were 4 weeks old upon arrival. Mice were housed under standard housing conditions: filter top cages, temperature 20–24 °C, 12 h light-dark cycle, and ad libitum access to animal chow and water.

### Macroscopic scoring of swelling

Arthritis development in each hind paw in IL-1Ra^–/–^ mice was macroscopically scored weekly or every 2 weeks using an arbitrary scoring system on a scale of 0 to 2 per paw as described previously [32]. The following scoring criteria were used: 0, no redness and swelling; 0.25, slight redness; 0.5, slight redness and swelling; 0.75–1, mild redness and swelling; 1.25–1.5, moderate redness and swelling; 1.75–2, severe redness and swelling. Only hind paws were scored as arthritis rarely develops in the fore paws.

### Serum cytokine and S100A8/A9 measurement

Blood was drawn from the retro-orbital plexus and collected in MiniCollect Serum Separator tubes (Greiner Bio-One). Serum concentrations of the cytokines IL-1β, IL-6, tumor necrosis factor (TNF), IL-17, IL-4, and interferon (IFN)-γ were determined using the Luminex multi-analyte technology on the Bio-Plex 100 system (Bio-Rad) in combination with the multiplex cytokine kit (Milliplex, Millipore, Amsterdam, the Netherlands). Serum was three-times diluted and sensitivity was >0.36 pg/ml. Serum S100A8/A9 concentrations were determined by an in-house sandwich ELISA specifically for mouse S100A8/A9 as described previously [23].

### Histological analysis of joint inflammation and damage

For assessment of joint inflammation and damage, total ankle joints were dissected and fixed in 4 % formalin for 4 days. Knees were decalcified in 5 % formic acid, dehydrated in a series of ethanol and embedded in paraffin. Sections of 7 μm were cut and stained with hematoxylin and eosin (H&E) for analysis of cell influx, bone erosion, and chondrocyte death, and Safranin O (SafO) staining for proteoglycan (PG) depletion and cartilage erosion. Each parameter was arbitrarily scored on a scale of 0 to 3 with steps of 0.25 by two independent observers in a blinded manner. Histological parameters were determined in the joints of the tibia and talus (tibio-talar joint) and of the talus and navicular bone (talo-navicular joint); three sections per ankle joint were scored and the mean score was determined.

### Immunohistochemistry

Tissue sections (7 μm) from formalin-fixed, paraffin-embedded ankle joints of IL-1Ra^–/–^ mice and BALB/c control mice were digested with proteinase-free chondroitinase ABC (0.25 units/ml in 0.1 M Tris-HCl, pH 8.0; Sigma-Aldrich) for antigen retrieval. Tissue sections were incubated overnight with rabbit anti-S100A8 and anti-S100A9 [23] or with rabbit anti-VDIPEN for staining of MMP-mediated cartilage destruction. Sections were then incubated with biotinylated horseradish peroxidase-conjugated goat anti-rabbit IgG (Dako) as a second antibody followed by incubation with avidin-streptavidin-peroxidase (Elite-kit, Vector). Peroxidase activity was assessed by staining with 3,3′-diaminobenzidine (DAB; Powervision DAB, Immunologic, Duiven, the Netherlands) in the presence of H_2_O_2_ and all sections were counterstained with hematoxylin for S100A8 and S100A9 staining and with orange G (2 %) for VDIPEN staining.

### In vivo optical imaging

To monitor local synovial S100A8 expression and activated MMPs in vivo, optical imaging was performed in 16-week-old IL-1Ra^–/–^ mice. Mice received an intravenous (i.v.) injection via a tail vein of the specific Cy7-labeled S100A8 polyclonal antibody (anti-S100A8-Cy7) or Cy7-labeled antibody of irrelevant specificity (Rabbit-IgG-Cy7) (2 nmol of Cy7 ~ 100 μg antibody) [23]. For MMP imaging, mice received an i.v. injection of 2 nmol Cy5.5-labeled AF489 (AF489-Cy5.5), a low-molecular weight MMP inhibitor that targets the active site of MMPs in vivo [33].

Twenty-four or 3 h post i.v. injection of anti-S100A8-Cy7 and AF489-Cy5.5, respectively, mice were anesthetized (2.5 % isoflurane/oxygen) and placed in the light-tight chamber and imaged with the IVIS Lumina (Caliper Life Sciences, Hopkinton, MA, USA) for 1 min. For imaging of the Cy7-labeled antibodies, excitation and emission wavelengths were set at 710 and 810–885 nm, respectively, and for AF489-Cy5.5 at 640 and 695–770 nm. Regions of interest were set on the inflamed ankle joint and muscle tissue (background). Signal-to-noise ratio (SNR) was calculated as SNR = mean fluorescent intensity of ankle/standard deviation of background signal.

### Statistical analysis

Nominal data are presented as mean values ± standard deviation, and ordinal data with box-and-whisker plot. Differences between groups were assessed by Student’s unpaired *t* test, or Mann-Whitney test when appropriate. Spearman rank correlation coefficients (rs) were calculated between serum levels of S100A8/A9 or pro-inflammatory cytokines and macroscopic and microscopic parameters for joint inflammation and destruction, and between fluorescent signal of in vivo imaging and macroscopic score for joint swelling. To determine the accuracy of serum S100A8/A9 as a biomarker for macroscopic joint swelling (score >0.5), receiver operating characteristic (ROC) curves were generated and the area under the curve (AUC) was calculated, where 0.5 represents random chance and 1.0 a perfect biomarker. Similarly, ROC and AUC were calculated to determine the accuracy of serum S100A8/A9 as biomarker for microscopic cell influx, bone erosion, cartilage erosion, PG depletion, and chondrocyte death (score >0.5). Statistical significance was set at *P* < 0.05 (two-tailed).

## Results

### Serum levels of S100A8/A9 correlate to macroscopic score for swelling in inflamed hind paws of IL-1Ra^–/–^ mice

**Fig. 1:**
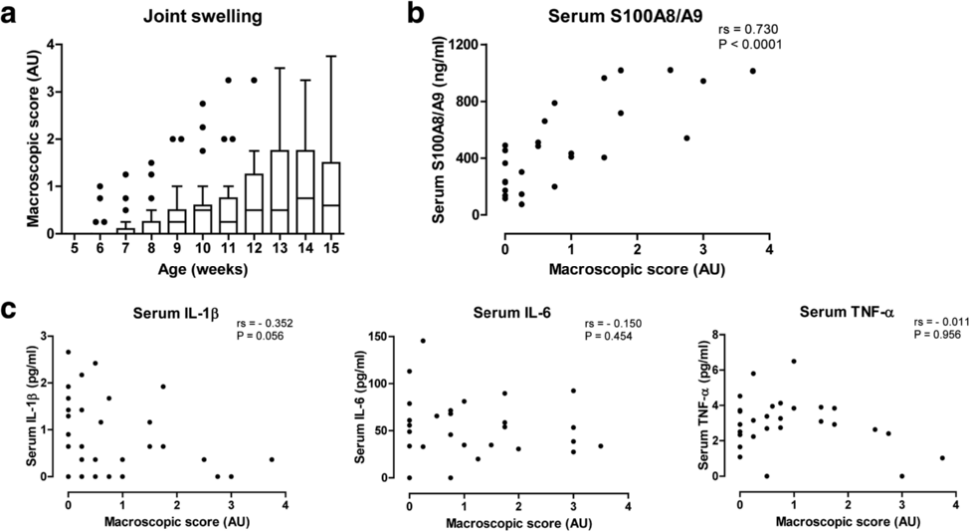
Serum S100A8/A9 levels correlate with macroscopic score for joint swelling. **a** Starting at week 6, IL-1Ra^–/–^ mice (*n* = 26) developed a spontaneous and highly heterogeneous joint swelling in the hind paws (combined score of left and right paw). **b** At end-point (week 15), serum levels of S100A8/A9 correlated to the combined macroscopic score for joint swelling. **c** Contrastingly, serum levels of the pro-inflammatory cytokines IL-1β, IL-6, and TNF did not correlate with the combined macroscopic score for joint swelling at week 15. *IL* interleukin, *IL-1Ra* interleukin-1 receptor antagonist, *TNF* tumor necrosis factor

IL-1Ra^–/–^ mice (*n* = 26) developed a spontaneous and heterogeneous joint swelling in the hind paws starting at week 6, reaching a combined median joint swelling in both ankle joints of 0.6 on a scale of 0 to 4 with a disease incidence of 70 % at week 15 (Fig. 1a). Serum levels of S100A8/A9 were higher in mice with increased joint swelling and correlated significantly with the macroscopic score for joint swelling at week 15 (rs = 0.730, *P* < 0.0001; Fig. 1b). Moreover, at week 15, S100A8/A9 levels were able to discriminate low joint swelling (score 0–0.5) from mild and severe swelling (score 0.75–4) with high accuracy (AUC = 0.88, 95 % confidence interval (CI) 0.75–1.00; Additional file 1A). In contrast, serum levels of various pro-inflammatory cytokines did not correlate with joint swelling: IL-1β (rs = –0.352, *P* = 0.056), IL-6 (rs = –0.150, *P* = 0.454), and TNF (rs = –0.011, *P* = 0.956) (Fig. 1c). Serum levels of the T cell-related cytokines IL-17 (rs = 0.304, *P* = 0.102), IL-4 (rs = –0.004, *P* = 0.983), and IFN-γ (rs = –0.019, *P* = 0.921) also did not correlate with joint swelling (Additional file 2A).

### Early serum levels of S100A8/A9 are prognostic for the development of joint swelling in IL-1Ra^–/–^ mice

**Fig. 2:**
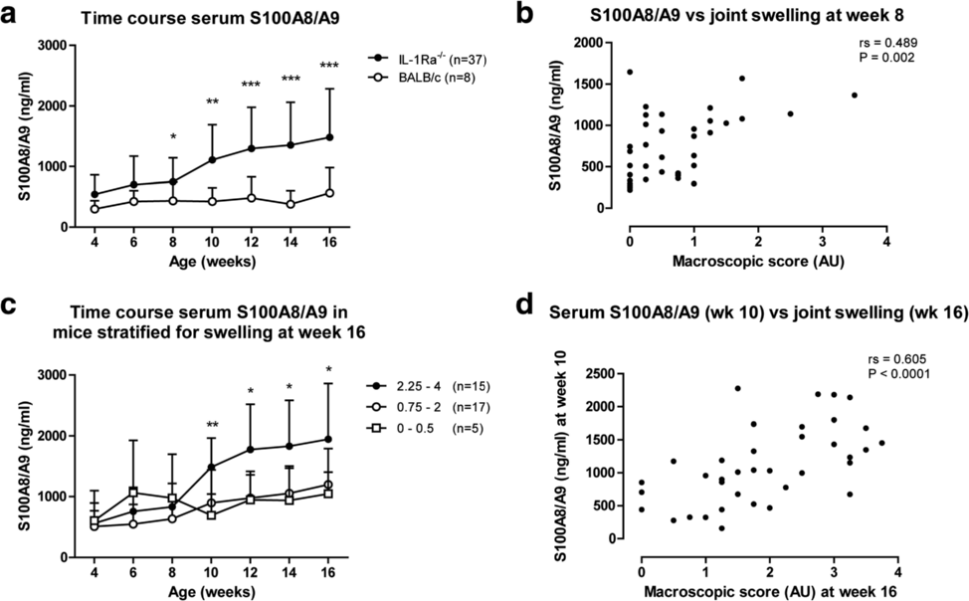
Early serum S100A8/A9 levels are prognostic for development of increased joint swelling. **a** Serum levels of S100A8/A9 in IL-1Ra^–/–^ mice (*n* = 37) were increased compared to BALB/c mice (*n* = 8) at week 8 of age (*P* = 0.029) and at weeks 10, 12, 14, and 16. **b** At week 8, serum levels of S100A8/A9 in IL-1Ra^–/–^ mice correlated with the combined macroscopic score for joint swelling, and continued to be correlated with joint swelling at weeks 10, 12, 14, and 16 (data not shown). **c** IL-1Ra^–/–^ mice were stratified for macroscopic joint swelling at week 16 in severe (score 2.25–4), mild (score 0.75–2), and low (0–0.5) joint swelling. Serum S100A8/A9 levels were increased in severe arthritic mice as early as week 10 compared to mild (*P* = 0.004) and low arthritic mice (*P* = 0.009). Serum levels of S100A8/A9 in severe arthritic mice continued to be increased compared to mild and low arthritic mice at weeks 12, 14, and 16. **d** Serum levels of S100A8/A9 at week 10 correlated with joint swelling at week 16, indicating that early serum S100A8/A9 levels are prognostic for disease outcome at a later stage. *P < 0.05, **P < 0.01, ***P < 0.001. *IL-1Ra* interleukin-1 receptor antagonist

To investigate whether serum levels of S100A8/A9 are prognostic for disease outcome, serum S100A8/A9 levels were measured in IL-1Ra^–/–^ mice (*n* = 37) every 2 weeks starting at week 4 when arthritis has not developed yet, and compared to levels in age-matched BALB/c control mice (*n* = 8). S100A8/A9 levels in IL-1Ra^–/–^ mice gradually increased during development and were significantly increased compared to control mice at 8 weeks of age (*P* = 0.029; Fig. 2a). From that time point on, serum S100A8/A9 levels correlated with the macroscopic score for joint swelling (rs = 0.489, *P* = 0.002; Fig. 2b) throughout disease development.

To investigate the potential of S100A8/A9 as a prognostic biomarker, we investigated whether increased S100A8/A9 serum levels at early time points were associated with increased joint swelling at the 16-week end point. As development of arthritis in IL-1Ra^–/–^ mice is variable, mice were stratified at end point (week 16) into three groups, i.e., mice with low (score 0–0.5), mild (score 0.75–2), and severe macroscopic swelling (score 2.25–4). Next, serum S100A8/A9 levels in the three groups were investigated throughout disease development. Whereas serum S100A8/A9 levels between low and mild arthritic mice did not differ, severe arthritic mice showed increased serum S100A8/A9 levels compared to both low and mild arthritic mice at week 10 (*P* = 0.009 and *P* = 0.004, respectively), indicating that high serum S100A8/A9 levels at week 10 are prognostic for severe macroscopic swelling (score 2.25–4) at week 16 (Fig. 2c). The prognostic value of serum S100A8/A9 in IL-1Ra^–/–^ mice at week 10 is further strengthened by its correlation with the macroscopic score for swelling at week 16 (rs = 0.605, *P* < 0.0001; Fig. 2d). Furthermore, while at weeks 4, 6, and 8 serum S100A8/A9 levels were not able to predict severe joint swelling (score 2.25–4) at week 16 (AUC = 0.52, 0.61, and 0.61, 95 % CIs 0.32–0.71, 0.42–0.79 and 0.43–0.79, respectively; Additional file 1B), serum S100A8/A9 levels were an accurate prognostic biomarker at weeks 10, 12, and 14 (AUC = 0.82, 0.88, and 0.81, 95 % CIs 0.68–0.96, 0.77–0.99, and 0.67–0.95, respectively; Additional file 1B).

### Serum S100A8/A9 correlates with histological parameters of inflammation and local S100A8 and S100A9 levels in the synovium of inflamed ankle joints of IL-1Ra^–/–^ mice

**Fig. 3:**
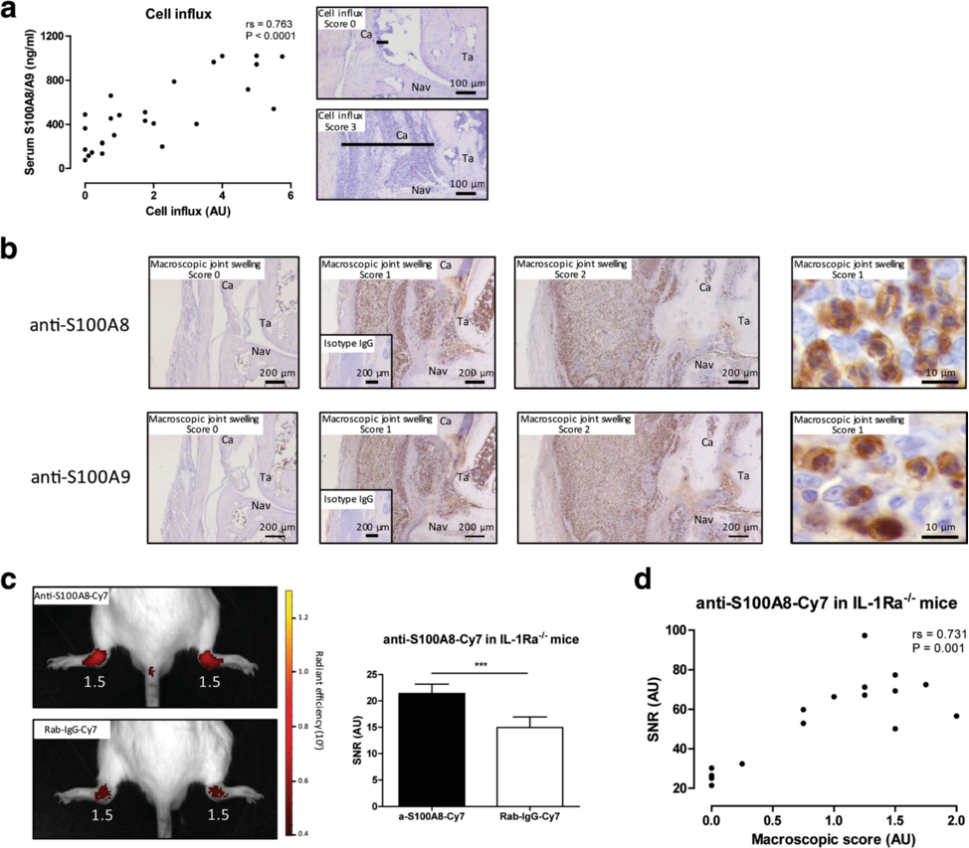
Serum S100A8/A9 levels correlate with histological parameters of inflammation and local synovial S100A8 and S100A9 levels. **a** Increased joint swelling was associated with increased thickening of the synovium (*black line*) and serum levels of S100A8/A9 correlated with the cell influx in the synovium of 16-week-old IL-1Ra^–/–^ mice. **b** IL-1Ra^–/–^ mice with increased macroscopic score for joint swelling showed a clear increased S100A8 and S100A9 expression, with similar distribution, in the infiltrating cells of the ankle joint. Expression of S100A8 and S100A9 within the inflamed ankle joint of IL-1Ra^–/–^ mice is co-expressed in monocytes and neutrophils. Isotype control IgG staining for S100A8 and S100A9 staining showed no staining in ankle joint sections of arthritic IL-1Ra^–/–^ mice (inserts). **c** Intravenous injection of polyclonal anti-S100A8-Cy7 in 16-week-old arthritic IL-1Ra^–/–^ mice (*n* = 6) led to a significantly increased fluorescent signal in the ankle joints compared with mice injected with irrelevant Rab-IgG-Cy7 (*n* = 6) (*P* = 0.0002) (macroscopic score for joint swelling in *white*). **d** Anti-S100A8-Cy7 targeting was imaged in ankle joints of IL-1Ra^–/–^ mice with various degrees of joint swelling and the observed fluorescent signal correlated with the macroscopic score for joint swelling. ***P < 0.001. *Ca* calcaneus, *Ig* immunoglobulin, *IL-1Ra* interleukin-1 receptor antagonist, *Nav* navicular bone, *Ta* talus

We next investigated whether serum levels of S100A8/A9 at 16 weeks, apart from macroscopic score for joint swelling, also correlated with microscopic parameters for joint inflammation. Synovial cell influx was determined in the tibio-talar and talo-navicular joints using an arbitrary scale of 0 to 3, and the combined score of both ankle joints was determined. Ankle joints of 16-week-old arthritic IL-1Ra^–/–^ mice showed increased amounts of infiltrated inflammatory cells (≈60 % neutrophils and 40 % monocytes/macrophage) in the synovium, which correlated closely with the macroscopic score of swelling (rs = 0.930, *P* < 0.0001; Additional file 2B) and serum levels of S100A8/A9 (rs = 0.763, *P* < 0.0001; Fig. 3a). Additionally, serum S100A8/A9 was able to discriminate increased cell influx (score >0.5) with a high accuracy (AUC = 0.93, 95 % CI 0.82–1.00; Additional file 1C).

A prominent expression of S100A8 and S100A9 within the activated synovium and infiltrating cells in the surrounding tissue of the ankle joints of 16-week-old IL-1Ra^–/–^ mice was visualized by immunohistochemistry using a specific antibody against murine S100A8 and S100A9 in serial sections (Fig. 3b). No expression of S100A8 or S100A9 was observed in the synovia of IL-1Ra^–/–^ mice with no swelling (score 0), whereas in mice with a macroscopic score for joint swelling of 1 and 2 a clear increased S100A8 and S100A9 expression was observed in the inflamed ankle joint (Fig. 3b). Expression of S100A8 and S100A9 within the inflamed ankle joint of IL-1Ra^–/–^ mice show a similar distribution and this is co-expressed in monocytes and neutrophils (Fig. 3b), indicating the inflamed joint as the source of increased serum S100A8/A9 levels in high arthritic IL-1Ra^–/–^ mice. No staining with isotype control IgG was found on ankle joint sections of arthritic IL-1Ra^–/–^ mice (inserts in Fig. 3b).

Since expression of both S100A8 and S100A9 is similar in ankle joints of arthritic IL-1Ra^–/–^ mice, we continued examining S100A8 only in the following experiments. To investigate whether local expression of S100A8 in high arthritic IL-1Ra^–/–^ mice could also be visualized in vivo we used a specific anti-S100A8 antibody coupled to Cy7. Targeting of anti-S100A8-Cy7 was assessed in 16-week-old IL-1Ra^–/–^ mice using optical imaging, and a fluorescent signal was observed in inflamed ankle joints (mean macroscopic score of 1.4 ± 0.3, *n* = 6; Fig. 3c). Specificity of the anti-S100A8-Cy7 signal was confirmed by comparison to the signal of an irrelevant rabbit-IgG-Cy7 in IL-1Ra^–/–^ mice with comparable joint swelling (mean macroscopic score of 1.3 ± 0.4, *n* = 6) which was significantly lower (*P* = 0.0002; Fig. 3c).

To further explore whether S100A8 imaging can be used as a biomarker tool to distinguish disease severity in IL-1Ra^–/–^ mice, anti-S100A8-Cy7 targeting was imaged in ankle joints of IL-1Ra^–/–^ mice with various degrees of joint swelling and showed a significant correlation with macroscopic score for joint swelling (rs = 0.731, *P* = 0.001; Fig. 3d). These data indicate that S100A8 in the inflamed joint of IL-1Ra^–/–^ mice can be visualized non-invasively by molecular imaging using anti-S100A8-Cy7, and may be a relevant biomarker tool to assess disease severity.

### Serum S100A8/A9 correlates with histological parameters of bone erosion and cartilage damage in the inflamed ankle joints of IL-1Ra^–/–^ mice

**Fig. 4:**
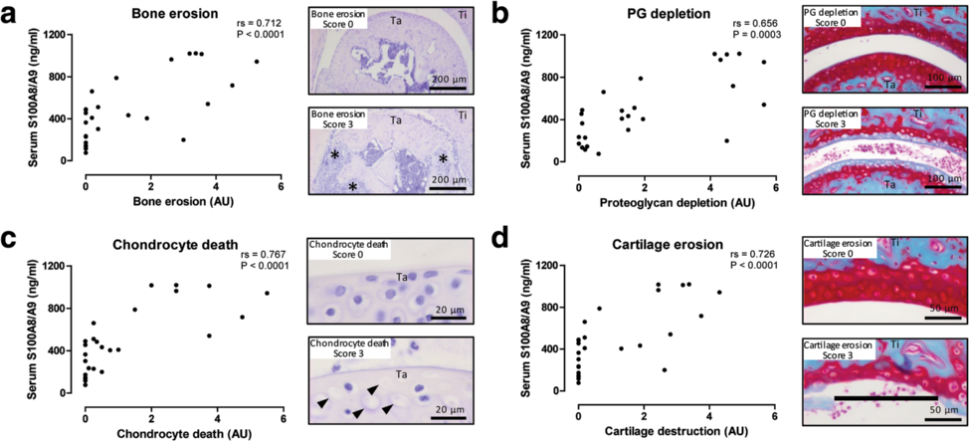
Serum S100A8/A9 levels correlate with histological parameters of bone erosion and cartilage damage. **a** Increased bone erosion (*) was observed in arthritic IL-1Ra^–/–^ mice, which correlated with serum levels of S100A8/A9. **b**–**d** Several parameters for cartilage damage were investigated. Proteoglycan depletion (**b**), chondrocyte death (**c**, *arrow heads*), and cartilage erosion (**d**, *black line*) were increased in arthritic IL-1Ra^–/–^ mice, and all these parameters correlated to serum levels of S100A8/A9. *Nav* navicular, *PG* proteoglycan, *Ta* talus, *Ti* tibia

Next, we studied the relationship between S100A8/A9 and joint destruction in 16-week-old IL-1Ra^–/–^ mice. First, we determined bone erosion on eight bone surface areas of the tibia, talus, and navicular bone. The combined mean bone erosion score of both ankle joints correlated with serum levels of S100A8/A9 in IL-1Ra^–/–^ mice (rs = 0.712, *P* < 0.0001; Fig. 4a), and at week 16 serum S100A8/A9 was able to discriminate increased bone erosion (score >0.5) with high accuracy (AUC = 0.87, 95 % CI 0.72–1.00; Additional file 1C).

Subsequently, various parameters for cartilage destruction were studied on the four cartilage surfaces of the tibio-talar and talo-navicular joints, and the combined mean score of both ankle joints was determined. All parameters correlated with serum levels of S100A8/A9, i.e., PG depletion (rs = 0.656, *P* = 0.0003; Fig. 4b), chondrocyte death (rs = 0.767, *P* < 0.0001; Fig. 4c), and cartilage erosion (rs = 0.726, *P* < 0.0001; Fig. 4d), and serum S100A8/A9 levels were able to discriminate for increased PG depletion, chondrocyte death, and cartilage erosion (score >0.5) with high accuracy (AUC = 0.84, 0.92, and 0.87, 95 % CIs 0.69–1.00, 0.81–1.00, and 0.72–1.00, respectively; Additional file 1C).

### Increased S100A8 expression is associated with increased MMP activity in the inflamed ankle joints of IL-1Ra^–/–^ mice

**Fig. 5:**
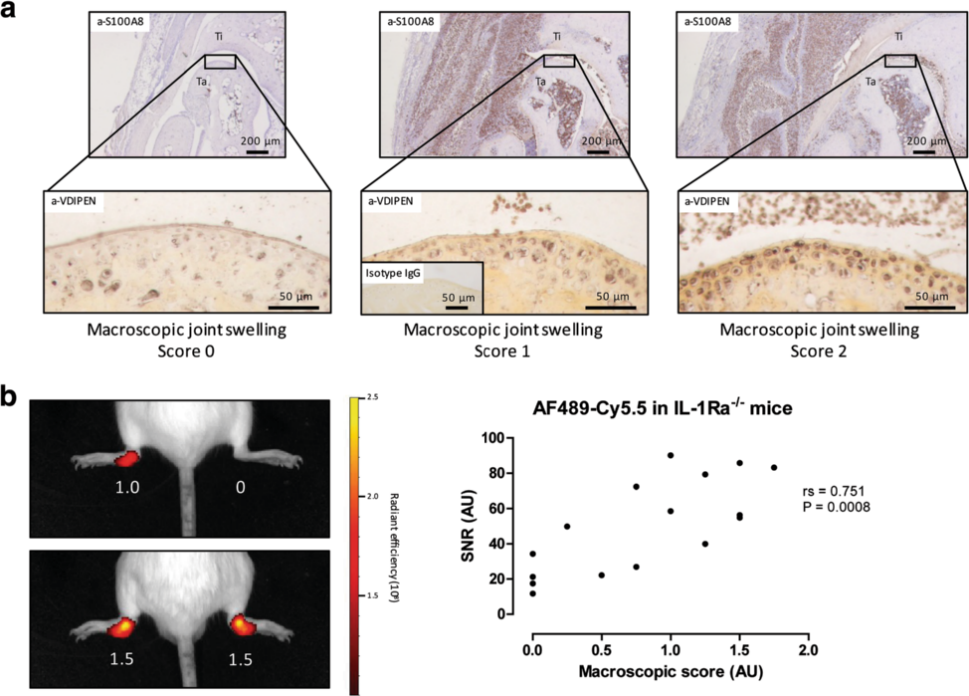
Increased S100A8 expression is associated with increased MMP activity in the inflamed ankle joints. **a** Increased expression of S100A8 in the inflamed ankle joints in 16-week-old IL-1Ra^–/–^ mice was associated with increased VDIPEN staining around the chondrocytes and cartilage matrix of the articular cartilage. Isotype control IgG staining for VDIPEN staining showed no staining in ankle joint sections of arthritic IL-1Ra^–/–^ mice (insert). **b** Intravenous injection of the MMP inhibitor AF-489 coupled to Cy5.5 led to an increased fluorescent signal in the inflamed ankle joints of arthritic IL-1Ra^–/–^ mice and the observed fluorescent signal correlated to the macroscopic score for joint swelling (macroscopic score for joint swelling in *white*). *Ig* immunoglobulin, *IL-1Ra* interleukin-1 receptor antagonist, *SNR* signal-to-noise ratio, *Ta* talus, *Ti* tibia

As MMPs are major enzymes involved in mediating cartilage destruction, we additionally investigated their link with the increased S100A8 levels that were observed in ankle joints of arthritic IL-1Ra^–/–^ mice. First, MMP-generated VDIPEN aggrecan neoepitopes were determined within the cartilage of ankle joint sections of arthritic IL-1Ra^–/–^ mice using immunohistochemistry. Increased MMP-mediated VDIPEN staining in the cartilage was associated with increased expression of S100A8 in the synovium, as shown by S100A8 and VDIPEN immunostaining in directly adjacent sections (Fig. 5a). VDIPEN neoepitopes were mainly found in the extracellular matrix surrounding the chondrocytes in the articular cartilage (Fig. 5a). In IL-1Ra^–/–^ mice with no joint swelling, little VDIPEN staining was visible around the chondrocytes, while in mice with increased joint swelling a gradual increase in VDIPEN staining was also observed in the extracellular matrix. In IL-1Ra^–/–^ mice with a maximum score for joint swelling of 2, all chondrocytes and the cartilage matrix were VDIPEN-positive (Fig. 5a), indicative of severe MMP-mediated cartilage breakdown. No staining with isotype control IgG was found on ankle joint sections of arthritic IL-1Ra^–/–^ mice (insert Fig. 5a).

Finally, to investigate the relationship between S100A8/S100A9 and MMP activity within the inflamed joints of IL-1Ra^–/–^ mice, we measured activated MMPs in IL-1Ra^–/–^ mice using molecular imaging. To this end, we used the low-molecular weight MMP inhibitor AF-489 which has been shown to bind to the active sites of several gelatinases and collagenases involved in cartilage breakdown, i.e., MMP-2, -9 and -13, with IC_50_ values in the nanomolar range [33]. Targeting of the MMP inhibitor AF489-Cy5.5 was clearly visible in inflamed ankle joints of IL-1Ra^–/–^ mice, which correlated with increased macroscopic swelling of the ankle joint (rs = 0.751, *P* = 0.0008; Fig. 5b ). Increased levels of active MMPs can be visualized in vivo by optical imaging and is associated with increased cartilage damage and VDIPEN neoepitopes in chondrocytes of IL-1Ra^–/–^ mice which are linked to increased levels of S100A8 expression in the synovium.

## Discussion

This study shows that the S100-DAMPs S100A8 and S100A9 can be used as biomarkers to assess disease activity in an experimental model of seronegative arthritis. Systemic and local levels of S100A8/A9, monitored by ELISA and in vivo imaging, correlate to parameters for joint inflammation and are prognostic for disease outcome at a later stage. Besides the potential as an inflammatory biomarker, S100A8/A9 may also serve as a biomarker for inflammatory joint destruction.

We here introduce the IL-1Ra^–/–^ mouse as an animal model for exploring new biomarkers in arthritides in which serum autoantibodies are not increased and not as a model for AS, PsA, or JIA, although these mice show some communalities with these human seronegative conditions. First of all, although serum levels of some autoantibodies are somewhat elevated in IL-1Ra^–/–^ mice compared to control BALB/c mice (i.e., RF against IgG, type II collagenase autoantibodies, and double-stranded DNA are increased 1.4-, 2.8- and 1.9-fold, respectively), these autoantibody levels did not correlate with disease severity, and even mice with low autoantibody levels were shown to develop arthritis [27]. In addition, IL-1Ra^–/–^ mice also display extra-articular inflammation, such as psoriasis [34] and aortitis [31, 35]. Another important communality between certain subsets of human seronegative arthritides and IL-1Ra^–/–^ mice is the involvement of increased IL-1 signaling, which is most apparent in sJIA patients [36, 37]. Beside increased IL-1 signaling, these patients also exhibit extraordinarily high levels of serum S100A8/A9 (a 44-times increase compared to healthy controls) suggesting a close relation between S100A8/A9 and IL-1β in inflammatory diseases.

While serum levels of S100A8/A9 in IL-1Ra^–/–^ mice correlated well with joint swelling and were able to discriminate for joint swelling (score >0.5) with high accuracy, serum levels of several key cytokines did not correlate with disease activity despite their functional role in joint pathology. The poor biomarker quality of these cytokines in IL-1Ra^–/–^ mice is in line with earlier observations where plasma levels of IL-1β and IL-6 in IL-1Ra^–/–^ mice remained unchanged compared to control BALB/c mice, while plasma levels of TNF were only moderately elevated [38]. Although IL-1β and TNF are major therapeutic targets in patients with seronegative arthritis, they are less suitable as serum biomarkers because of practical limitations, e.g., low serum levels and low thermal stability, which reduce accuracy of ex vivo measurements [39].

The search for more reliable biomarkers for seronegative arthritis has resulted in several serum proteins that are associated with disease activity in patients with seronegative arthritis: IL-6, IL-17, IL-23, VEGF, and MMP3 in SpA [6, 7, 40–43], and IL-6 and IL-18 in JIA [5, 36, 44], amongst others. Although these proteins correlate with certain clinical aspects of seronegative arthritis, a major problem remains the lack of specificity, and results are often inconsistent; moreover, these putative biomarkers still await validation in cohort studies. An alternative biomarker for seronegative arthritis is S100A8/A9, since it is released in high quantity during inflammation and it is highly stable (transition temperature >50 °C for human S100A8/A9) [45] and tolerates several freeze/thaw cycles of the sample without loss of human and murine S100A8/A9 levels (unpublished data). Additionally, S100A8/A9 is selectively released from early infiltrating phagocytes, thereby reflecting a local first-line response of the innate immune system in arthritis development.

Serum S100A8/A9 levels have already been implicated as a biomarker for disease activity and therapy response monitoring in seronegative arthritis. Serum levels of S100A8/A9 are increased and correlate with disease activity in AS [10], PsA [11, 26], and JIA [46, 47], and were significantly decreased after treatment with TNF-blockers. Additionally, high baseline serum S100A8/A9 levels in JIA are predictive of a good response to methotrexate and anti-TNF treatment [46, 47], whereas high serum levels of S100A8/A9 after complete remission can predict a relapse of inflammatory flares [12, 47, 48].

We show that, in IL-1Ra^–/–^ mice, serum S100A8/A9 levels are not only correlated with macroscopic joint swelling, but also with the influx of immune cells (mainly neutrophils and monocytes) in the inflamed joints. The high expression of S100A8 and S100A9 in these infiltrating cells indicates that the inflamed ankle joints are the source of the increased serum S100A8/A9 levels. Consequently, monitoring local expression of S100A8 (and S100A9) may be an even more specific biomarker tool to assess disease activity in human seronegative arthritis. In this study, we demonstrate that local expression of S100A8 in IL-1Ra^–/–^ mice could be monitored non-invasively by in vivo optical imaging and that the signal correlated with disease activity. A possible advantage of imaging locally produced S100A8 or S100A9 maybe the detection of sub-clinical inflammation, assuming local expression of S100A8 and S100A9 precedes the increases in serum S100A8/A9. Indeed, imaging of early S100A8 and S100A9 expression in inflamed joints of mice with collagen-induced arthritis (CIA), just after onset of the first CIA clinical signs, correlated strongly with disease outcome at a later stage [23], which may also apply to IL-1Ra^–/–^ mice.

Besides the potential as a biomarker for inflammation, S100A8/A9 may also serve as a biomarker for inflammatory joint destruction in seronegative arthritis. In this study, we show that serum S100A8/A9 levels are correlated with various microscopic parameters for bone and cartilage destruction in IL-1Ra^–/–^ mice. Not only are systemic levels of S100A8/A9 correlated with cartilage damage, but also local expression of S100A8 in the inflamed joint was associated with increased MMP-mediated cartilage damage, as demonstrated by the increased levels of the aggrecan neoepitope VDIPEN in the articular cartilage. In addition, we were able to non-invasively monitor increased levels of activated MMPs within the inflamed ankle joint by optical imaging using AF489-Cy5.5.

Experimental evidence shows a direct effect of S100A8 and S100A9 on processes involved in bone and cartilage damage, supporting the statement that these S100-DAMPs are a functional biomarker for joint destruction. A direct link between S100A9 and bone erosion has been described in the antigen-induced arthritis (AIA) model where S100A9^–/–^ mice developed less bone erosion which was accompanied by a reduction in the number of osteoclasts in the knee joints [49]. Furthermore, S100A8 was able to stimulate osteoclast formation and activity in vitro [49].

In addition, S100A8 and S100A9 are also directly involved in cartilage damage. S100A9^–/–^ mice with AIA show less cartilage destruction, i.e., PG depletion, chondrocyte death, and MMP activity, compared to wild-type mice [22]. Moreover, injection of recombinant S100A8 directly into a naive knee joint resulted in a rapid induction of synovial inflammation and cartilage PG depletion, which were accompanied by an increased expression of pro-inflammatory cytokines and MMPs [22]. A direct link for S100A8 and S100A9 on MMP expression in chondrocytes was further established by stimulation of murine and human chondrocytes with S100A8 and S100A9, which resulted in increased mRNA and protein expression of several MMPs and the generation of VDIPEN neoepitopes on the surface of the chondrocytes [19, 20].

Until now, only a few studies have investigated the biomarker potential of S100A8/A9 on joint damage in AS or SpA. Serum levels of S100A8/A9 have been found to correlate with radiographic features of arthritis in PsA [26], and were predictive of progression of radiographic damage of the spine and syndesmophyte formation in AS [25]. The correlation of serum S100A8/A9 with several aspects of bone and cartilage destruction in IL-1Ra^–/–^ mice, as described in the current study, further strengthens the concept of S100A8/A9 as a biomarker for inflammatory joint destruction in seronegative arthritis, and a biomarker for inflammation.

## Conclusions

The strong correlation between the expression of S100A8 and S100A9 with disease severity in IL-1Ra^–/–^ mice underlines the potential of serum S100A8/A9 as a diagnostic and prognostic biomarker in seronegative arthritis, not only for assessing inflammation but also for assessing inflammatory joint destruction. Indeed, several parameters for bone and cartilage damage correlated strongly to serum S100A8/A9 levels in IL-1Ra^–/–^ mice. Next to systemic levels, monitoring of local levels of S100A8 and S100A9 may be an important biomarker tool to assess joint inflammation and destruction as well, which can be inferred from the increased expression of S100A8 and S100A9 and in vivo imaging of S100A8 in the inflamed ankle joints hind paws of IL-1Ra^–/–^ mice.

## Additional files

Additional file 1: (A) Serum S100A8/A9 as a positive marker to predict joint swelling (macroscopic score >0.5) at week 16. (B) Serum S100A8/A9 at earlier time-points as a positive marker to predict severe joint swelling at week 16 (macroscopic score >2). (C) Serum S100A8/A9 as a positive marker to predict histological parameters for inflammation (cell influx), bone erosion, and cartilage damage (PG depletion, chondrocyte death, and cartilage erosion) (histological score >0.5) at week 16. (TIF 3155 kb)
Additional file 2: (A). Serum levels of the cytokines IL-17, IL-4, and IFN-γ did not correlate with the combined macroscopic score for joint swelling at week 15. (B) The combined macroscopic score for joint swelling correlated to the cell influx in the synovium of 16-week-old IL-1Ra^–/–^ mice. (TIF 1588 kb)

## Abbreviations

ABC: Avidin-biotin complex
ACPA: Anti-citrullinated protein antibodies
AIA: Antigen-induced arthritis
AS: Ankylosing spondylitis
AUC: Area under the curve
CI: Confidence interval
CIA: Collagen-induced arthritis
CRP: C-reactive protein
DAB: 3,3′-diaminobenzidine
DAMP: Damage-associated molecular pattern
ELISA: Enzyme-linked immunosorbent assay
ESR: Erythrocyte sedimentation rate
H&E: Hematoxylin and eosin
i.v.: Intravenous
IFN: Interferon
Ig: Immunoglobulin
IL: Interleukin
IL-1Ra: Interleukin-1 receptor antagonist
JIA: Juvenile idiopathic arthritis
MMP: Matrix metalloproteinase
MRP: Myeloid-related protein
PG: Proteoglycan
PsA: Psoriatic arthritis
RA: Rheumatoid arthritis
RF: Rheumatoid factor
ROC: Receiver operating characteristic
SafO: Safranin O
sJIA: Systemic onset juvenile idiopathic arthritis
SNR: Signal-to-noise ratio
SpA: Spondyloarthritis
TLR: Toll-like receptor
TNF: Tumor necrosis factor
