# Supplementary figures and images for: S100A8/A9, a potent serum and molecular imaging biomarker for synovial inflammation and joint destruction in seronegative experimental arthritis
Source: Arthritis Res Ther. 2016 Oct 24;18:247. doi: 10.1186/s13075-016-1121-z (PMC5078998; doi:10.1186/s13075-016-1121-z)

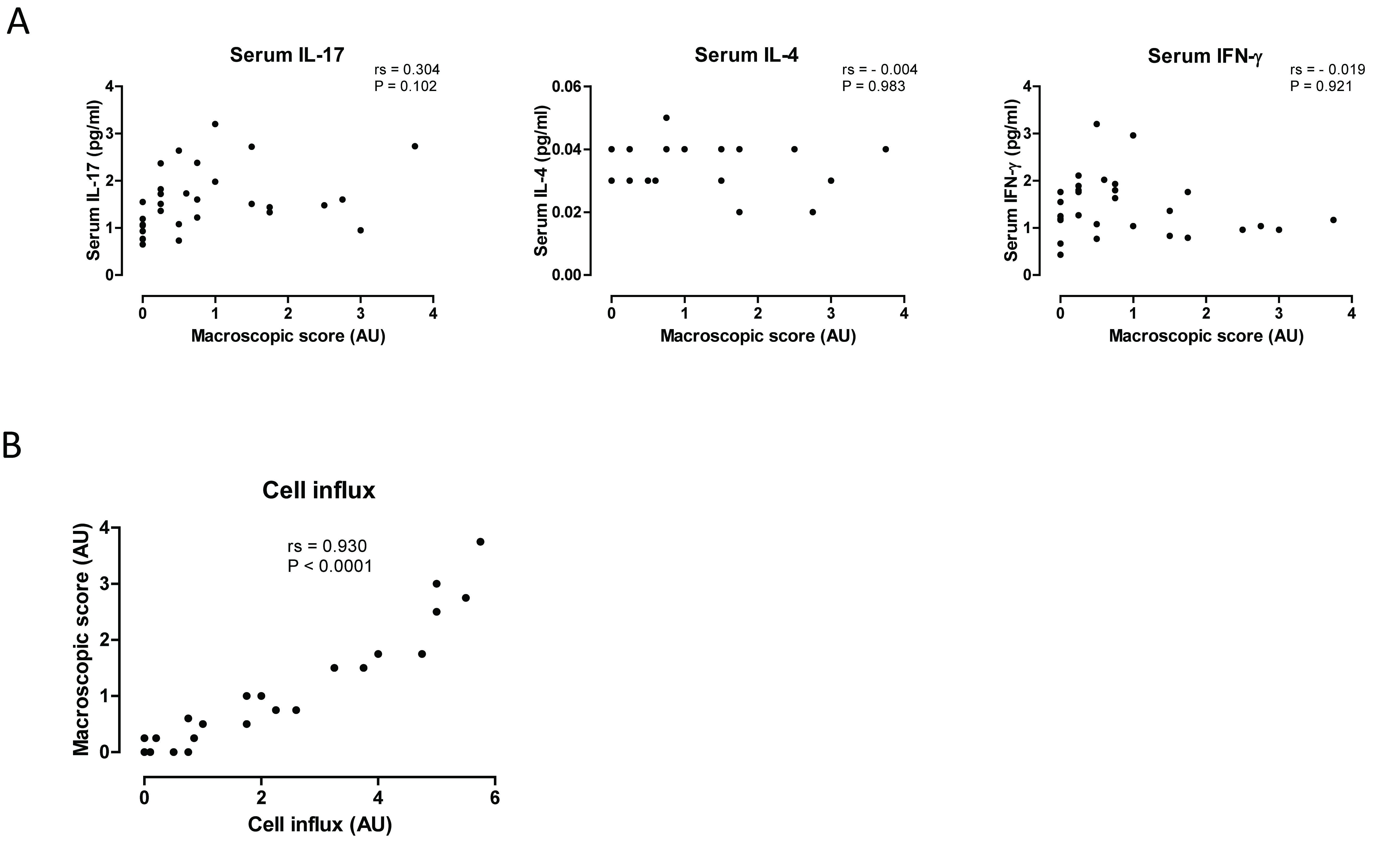

Supplement: Additional file 2: — (A). Serum levels of the cytokines IL-17, IL-4, and IFN-γ did not correlate with the combined macroscopic score for joint swelling at week 15. (B) The combined macroscopic score for joint swelling correlated to the cell influx in the synovium of 16-week-old IL-1Ra–/– mice. (TIF 1588 kb) [file 13075_2016_1121_MOESM2_ESM.tif]
